# Prevalence and interconnectedness of delirium, dementia, and frailty pathways in clinical settings: a survey of geriatricians across Europe

**DOI:** 10.1007/s41999-025-01375-w

**Published:** 2025-12-13

**Authors:** Mary Faherty, Aoife O’Connor, Catriona Curtin, Enrico Brunetti, Mario Bo, Alessandro Morandi, Antonio Cherubini, Massimiliano Fedecostante, Maria Cristina Ferrara, Alessandra Coin, Susan D. Shenkin, Pinar Soysal, Giuseppe Bellelli, Suzanne Timmons

**Affiliations:** 1 Centre for Gerontology and Rehabilitation, University College Cork, Cork, Ireland; 2 Mercy University Hospital, Cork, Ireland; 3 Section of Geriatrics, Department of Medical Sciences, University Hospital Città Della Salute E Della Scienza, Turin, Italy; 4 Department of Clinical and Experimental Science, University of Brescia, Brescia, Italy; 5 Azienda Speciale Cremona Solidale, Cremona, Italy; 6 Vall d’Hebrón Institute of Research, Barcelona, Spain; 7 Accettazione Geriatrica e Centro Di Ricerca Per L’Invecchiamento, IRCCS INRCA, Ancona, Italy; 8 Department of Clinical and Molecular Sciences, Università Politecnica Delle Marche, Ancona, Italy; 9 School of Medicine and Surgery, University of Milano-Bicocca, Milan, Italy; 10 Geriatrics Unit, Azienda Ospedale – Università Padova, Department of Medicine, University of Padova, Padua, Italy; 11 Ageing and Health Research Group, and Advanced Care Research Centre, Usher Institute, University of Edinburgh, Edinburgh, Scotland, UK; 12 Department of Geriatric Medicine, Faculty of Medicine, Bezmialem Vakif University, Istanbul, Türkiye; 13 School of Medicine and Surgery, University of Milano-Bicocca and Acute Geriatric Unit, IRCCS Foundation “San Gerardo Dei Tintori”, Monza, Italy

**Keywords:** Delirium, Dementia, Frailty, Health service, Care pathway

## Abstract

**Purpose:**

To explore the prevalence, overall and in different clinical settings, and interconnectedness, of delirium, dementia, and frailty clinical pathways across Europe.

**Methods:**

An online anonymous survey was distributed via the mailing list of the European Geriatric Medicine Society (EuGMS), national member groups and the authors’ professional networks, targeting geriatricians, or trainees in their final 2 years of specialist geriatric training, working in a hospital, rehabilitation, post-acute care or residential setting in a European country. Quantitative data were summarized using descriptive statistics and frequency distributions. Inductive content analysis was used to interpret open-text questions.

**Results:**

The 240 respondents were predominantly female (63%), with a 6:1 consultant to trainee ratio and marked underrepresentation of Eastern Europe. Integrated care pathways (current or in-development) for delirium, dementia, or frailty are reported in 48–78% of settings. Dementia and delirium pathways are common except in radiology, neurosurgery, and operating/recovery settings. Frailty pathways are less common overall, and specific frailty staff are less common than dementia or delirium staff. Dementia pathways commonly incorporate delirium screening (76%) and prevention (73%), but less commonly frailty screening (61%). Similarly, delirium pathways often provide guidance on formal dementia diagnosis (62%) but less than half incorporate frailty screening/assessment (46%). Notably, only 19% of delirium pathways differentiate between managing delirium and delirium-superimposed-on-dementia (DSD). Frailty pathways frequently incorporate cognitive assessment (81%) and delirium screening/assessment (75%), but only 57% incorporate delirium prevention.

**Conclusion:**

Dementia and delirium pathways are more common and more integrated and inclusive of each other than frailty pathways. More unified approaches could maximize the value of staff time, reduce duplications, and avoid a siloed approach to the care of older people.

**Supplementary Information:**

The online version contains supplementary material available at 10.1007/s41999-025-01375-w.

## Key summary points

**Aim:**

To explore the prevalence, overall and across specific clinical areas, of integrated care pathways for delirium, dementia, and frailty, and their interconnectedness, in European clinical settings.

**Findings:**

Integrated care pathways for delirium, dementia, and frailty are variably implemented across clinical settings in Europe. While dementia and frailty pathways frequently include delirium-related assessments, frailty screening in dementia and delirium pathways is low, and the differentiation between the management of delirium and delirium-superimposed-on-dementia is limited.

**Message:**

There is a need for greater integration of clinical care delivery to prevent fragmented approaches to the geriatric syndromes of delirium, dementia, and frailty.

**Supplementary Information:**

The online version contains supplementary material available at 10.1007/s41999-025-01375-w.

## Introduction

‘Integrated care pathways’ (ICPs), also known as clinical care pathways, clinical pathways, care pathways, critical pathways, care models, and care maps [1–3] have been defined as “structured multidisciplinary care plans which detail essential steps in the care of patients with a specific clinical problem” [1, p.133]. Others have built on this definition, highlighting various aspects including the role of ICPs in translating guidelines or evidence into local procedures; confirming detailed steps can take the form of a plan, pathway, algorithm, guideline, protocol, or other list of actions; calling for specific timeframes or criteria-based progression; and broadening the focus from a specific clinical problem to also include a procedure or episode of healthcare in a specific population [3, 4].

ICPs have several benefits. A systematic review (SR) and meta-analysis (MA) of 27 studies found that ICPs reduced in-hospital complications (odds ratio [OR] 0.58) and improved documentation (OR 11.95) [5]. Care processes organized by ICPs can lead to better coordination of care and follow-up of care [6]. A SR of 26 studies found low-quality evidence that ICPs lead to improved teamwork [7]. However, weaknesses have also been identified, including an increased documentation burden for clinicians and the risk of treating all patients homogeneously [8], with a UK care pathway being withdrawn when media claims it contributed to poor care [9].

While the use of ICPs across European countries has been steadily increasing [10], a 2005 survey across 17 European countries estimated average ICP use of 18% [8]. ICP use is most prevalent in teaching hospitals, acute hospital trusts, and rehabilitation centers [2]. Adoption in geriatric medicine is unclear, although a review of 207 ICP evaluations found only two pertained to geriatric medicine, with both for dementia care [11].

Delirium, dementia, and frailty are interlinked syndromes. Dementia is a progressive condition marked by a substantial decline from previous levels of cognitive performance in one or more cognitive domains that causes disability [12]. Delirium, characterized by acute and fluctuating disturbances of consciousness, cognitive function, or perception [13], is a known risk factor for dementia, and vice versa [14, 15]. Frailty is an age-related syndrome characterized by declining physiologic reserves and function [16] that also has clear associations with dementia [17], with frail older people more likely to have dementia [18]. Frailty and delirium are also interconnected [19], with a delirium relative risk of 1.66–2.96 for hospitalized older adults with frailty [20, 21]. The co-occurrence and links between delirium, dementia, and frailty imply that each should be assessed with due consideration of the other two syndromes [22].

Some European countries have developed national ICPs for these syndromes. The Health Service Executive in Ireland has developed ICPs for dementia and delirium [23]. The suite of resources includes three pathways and two algorithms which link to each other. However, frailty is not specifically mentioned, apart from being a risk factor for delirium [23]. Italy has produced national guidance on ICPs for people with dementia, although a survey reported only a moderate level of compliance on rollout [24]. Scotland’s delirium management pathway recognizes dementia and frailty as risk factors for delirium [25]. The NHS Rightcare Frailty toolkit in England prioritizes recognizing delirium, including delirium-superimposed-on-dementia (DSD). It recommends recognizing cognitive impairment using a standardized tool, with onward referral to a specialist service if required [26]. However, the implementation of ICPs for dementia, delirium, and frailty varies across European countries and settings, in part reflective of large differences in the approach and organization of health and social care of older people [27].

By examining data provided by geriatricians working in clinical settings in European countries, this study aims to assess the prevalence and interconnectedness of ICPs for delirium, dementia, and frailty, in hospital, rehabilitation, post-acute care and residential settings across Europe.

## Methods

### Study design

A quantitative design was employed, with participants completing an anonymous online survey. Participation in this study was voluntary.

### Survey design

The ICP questions comprised the final section of a broader 106-item survey of European geriatricians’ perceptions on frailty, delirium, and dementia, created de novo by members of the EuGMS SIG in Delirium, supported by the Dementia SIG [22]. The ICP section of the survey, consisting of 19 items, required approximately 10 min to complete. Availability was via an embedded link to a project website hosted by University College Cork, Ireland. Respondents were requested to provide answers in English only. ICP specific questions formed the final part of the survey (Online Resource 3) and comprised 14 quantitative and 5 qualitative questions on i) dementia care pathways, ii) delirium care pathways, iii) frailty care pathways, iv) clinical site staffing, and v) areas for improvement. The survey was piloted in English with two academic psychologists and five geriatricians who had not been involved in the survey development. This pilot data was not included in the analysis. Minor changes were made to item wording to improve clarity or aid question routing, and a definition of a clinical care pathway was added. The survey was then translated into 11 European languages.

### Sampling and recruitment

Eligible participants worked in a European hospital, rehabilitation, post-acute care, or residential setting, and satisfied one of the following criteria i) fully qualified (temporary or permanent) consultant geriatrician (currently or within last 12 months), or ii) geriatric trainee in final 2 years of specialist (higher) geriatric training, or iii) geriatrician retired less than 3 years from working in any European country. Participation was open to all ages and genders, and geriatricians were not required to have specific expertise in delirium, dementia, or frailty in their clinical practice. General physicians with a special interest in geriatric medicine were eligible in countries where geriatric medicine was not a national specialty. Participants with no clinical work were excluded.

Snowball sampling was used for recruitment. All members of the European Geriatric Medicine Society (EuGMS) were issued the survey and asked to respond and share it with colleagues (including non-EuGMS members). Members of the EuGMS Special Interest Groups (SIG) in Dementia, Delirium, and Frailty also promoted the survey through their respective national geriatric professional organizations.

### Data collection and analysis

Data were collected from September 2023 to June 2024 via a Qualtrics survey form.

Quantitative data were analyzed in Excel, with descriptive statistics presented as the number or percentage of participants endorsing a particular response, and frequency distributions. Open-text questions were analyzed using inductive content analysis, a commonly used method applied to open data to reduce and group information into concepts or categories [28].

## Results

### Demographic characteristics of respondents

**Fig. 1:**
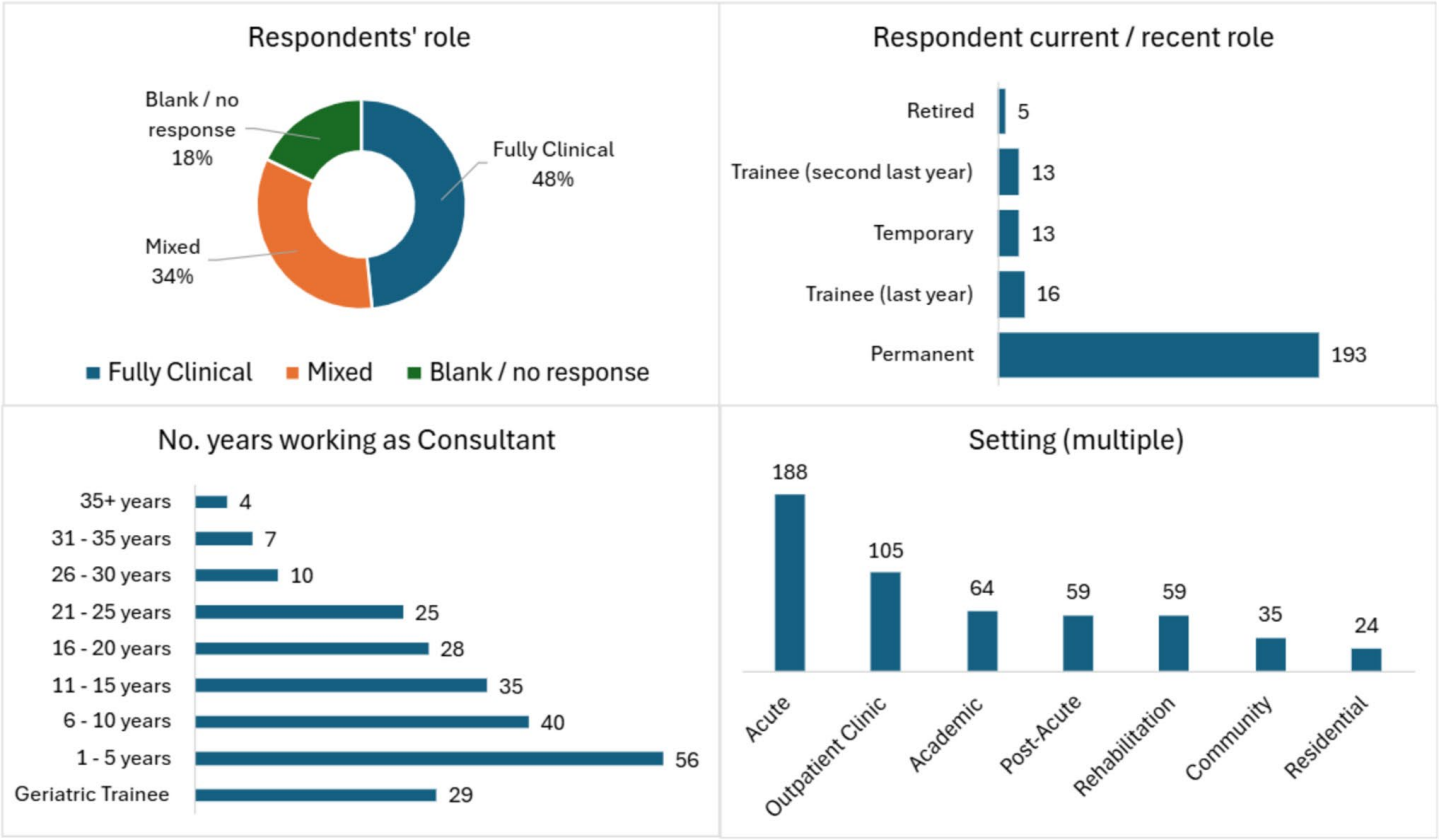
Participant demographics

While 440 participants took part in the broader survey, 277 respondents assessed their eligibility for the specific ICP portion of the broader survey, of whom 240 satisfied the requirements, i.e., they were familiar with working in a hospital, rehabilitation, post-acute, or residential setting. Participants spanned 27 countries with 1–31 responses per country (median = 7 and average = 9 responses per country). Twelve countries accounted for almost 80% of respondents (ref. Online Resource 1). Northern Europe was well represented (*n* = 88), followed by Southern Europe (*n* = 76) and Western Europe (*n* = 64), with a notable underrepresentation of Eastern Europe (*n* = 12). Where disclosed (*n* = 236), 63% of respondents were female. Professional status comprised 84% fully qualified consultants, 15% geriatric trainees, and 1% retired. Where indicated (*n* = 197), 59% were fully clinical, while 41% had a mixed clinical-academic role. The consultants had mixed duration of experience at that level: 47% had 1–10 years and 53% had 11 + years. Most respondents worked in an acute or outpatient setting (Fig. 1).

### Specific care pathways

**Fig. 2:**
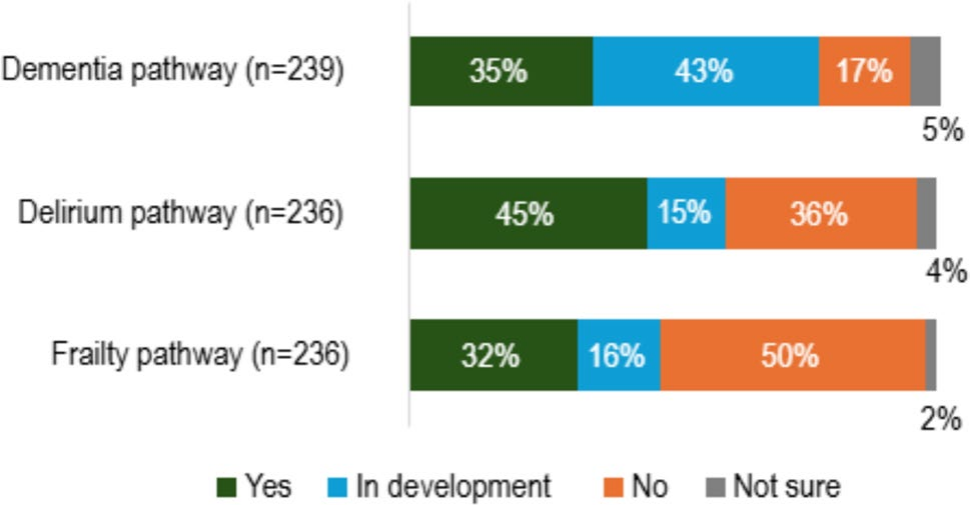
Prevalence of specific care pathways for frailty, delirium, and dementia at respondents’ main clinical site (excludes 1–4 blank responses)

To assess the prevalence of specific ICPs, participants were asked if their clinical site had a pathway for frailty, delirium, and dementia, respectively (Fig. 2). Delirium pathways were also more likely to be in existence rather than in-development. Given the limited respondents for some countries, reporting pathway prevalence at a country level would be of limited use. However, it is worth noting regional differences. Pathways (in existence or development) for dementia, delirium, and frailty, respectively, were less commonly reported in Eastern Europe (5,4,5 out of 12) and Southern Europe (33,40,29 out of 76) compared to Western Europe (37,44,30 out of 64) and Northern European countries (49,54,49 out of 88).

### Dementia care pathways

**Fig. 3:**
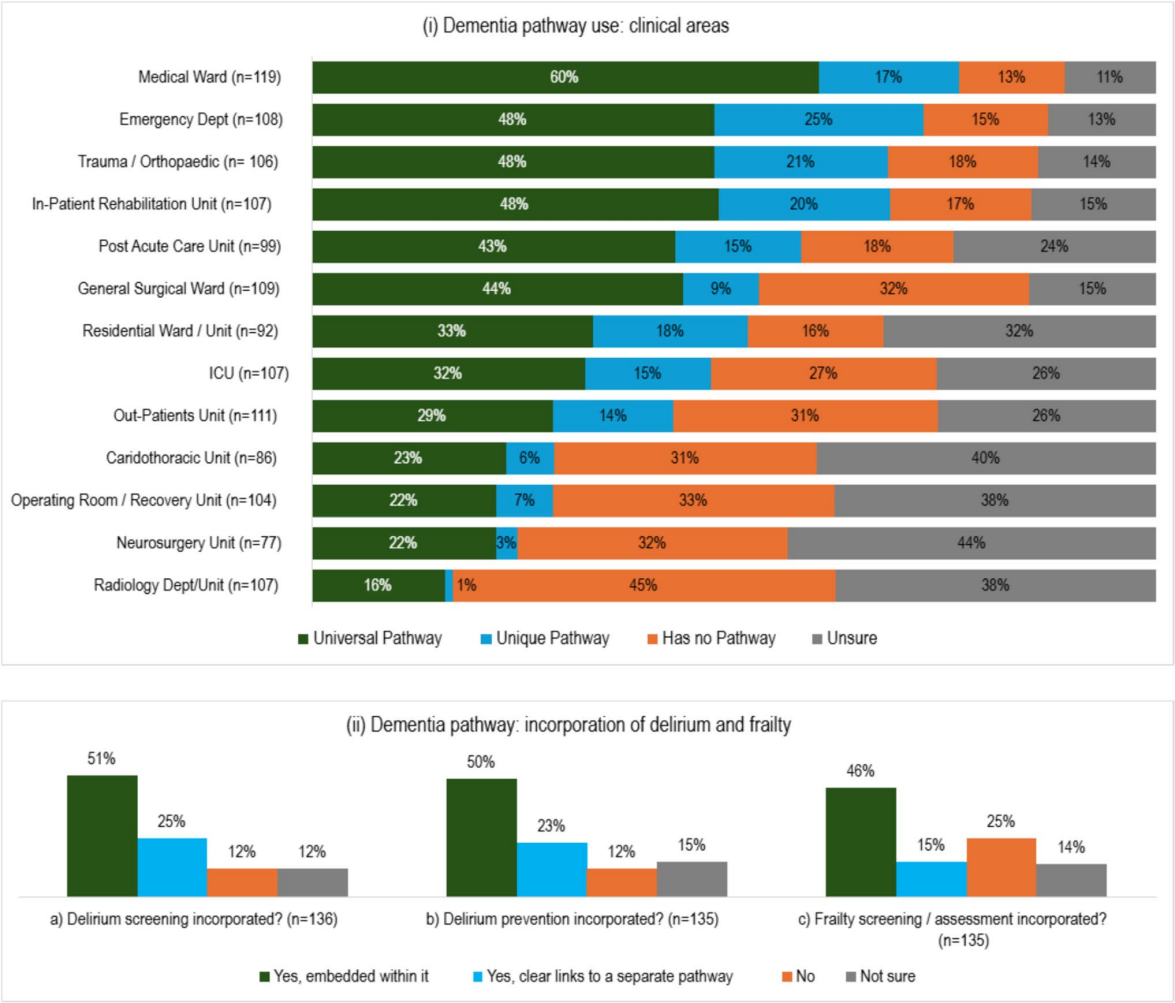
Dementia pathways: **i** prevalence within different clinical areas; **ii** incorporation of delirium (screening/assessment and prevention) and frailty screening/assessment

Respondents were asked to identify which areas in their clinical site have a dementia pathway in place or development (Fig. 3). Responses (*n* = 77–119) show the clinical units most likely to have a universal dementia pathway were medical wards (60%), followed by EDs, inpatient rehabilitation units, and trauma/orthopedic units (40%). A unique pathway for a clinical area was most common in EDs (25%), or trauma/orthopedic (21%) and inpatient rehabilitation (20%) units. Respondent uncertainty on the existence of dementia pathways within clinical sites varied from 11–44%.

Respondents whose clinical site had a dementia pathway (or a dementia pathway in an advanced stage of development) were asked about the integration of delirium and frailty within this pathway (Fig. 3). Respondents (*n* = 135 or 136) confirmed that dementia pathways incorporated delirium screening in 76% of cases, either embedded within the dementia pathway (51%) or clearly linked to a separate pathway (25%). Incorporation of delirium prevention was common (73%), most often embedded within the dementia pathway (50%). Just over one in ten respondents reported that their dementia pathway(s) had no delirium screening or prevention (12%). Frailty screening was also often embedded (46%) and occasionally linked to a separate pathway (15%).

### Delirium care pathways

Delirium care pathways were often specific for the clinical area, with unique pathways most common in medical units (23%), ICU (21%), EDs, trauma/orthopedic units, and inpatient rehabilitation units (all 19%). A universal delirium pathway was most frequently reported for medical units (60%) and EDs (51%). The prevalence of having no delirium pathway was highest for radiology (43%) and outpatients (39%), but also, surprisingly, operating room/recovery units (29%). Similar to the situation for dementia pathways, respondents were often unsure if a delirium pathway existed in radiology departments, operating room/recovery units, cardiothoracic and neurosurgical units (41–47%).

**Fig. 4:**
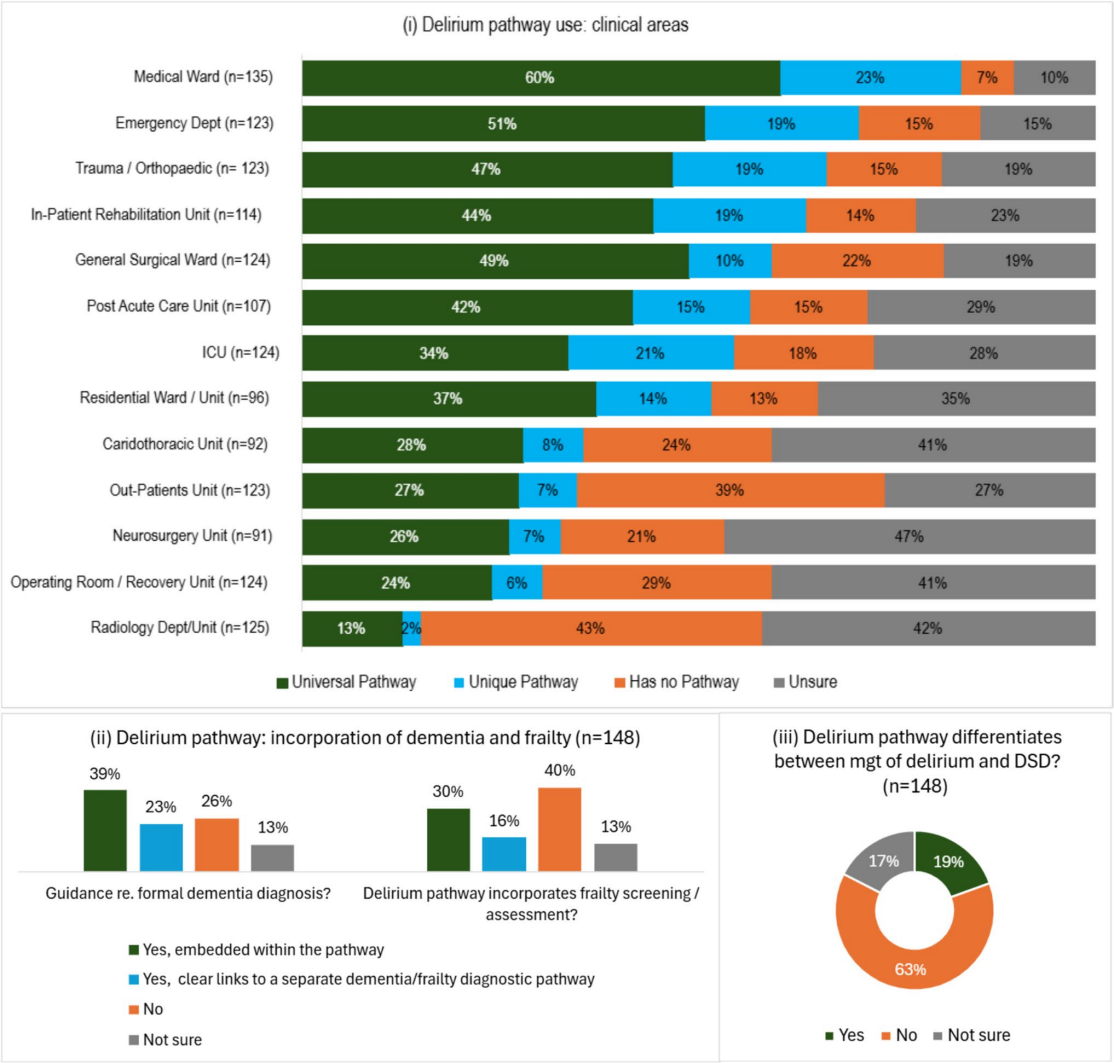
Delirium pathways: **i** prevalence across different clinical areas; **ii** incorporation of dementia and frailty assessment; **iii** differentiation between management of delirium in isolation and delirium-superimposed-on-dementia (DSD)

Respondents whose clinical site had a delirium pathway (either existing or at an advanced stage of development) were asked if the pathway incorporated directions or guidance for obtaining a formal dementia diagnosis where a patient was suspected to have underlying undiagnosed dementia. Overall, 62% of respondents (92 of 148) said dementia directions or guidance was incorporated, either embedded within the delirium pathway (39%) or clearly linked to a separate diagnostic pathway (23%). Delirium pathways less often included frailty screening and assessment (46%; Fig. 4). Differentiation between the management of delirium and delirium-superimposed-on-dementia (DSD) was rare (19%).

### Frailty care pathways

**Fig. 5:**
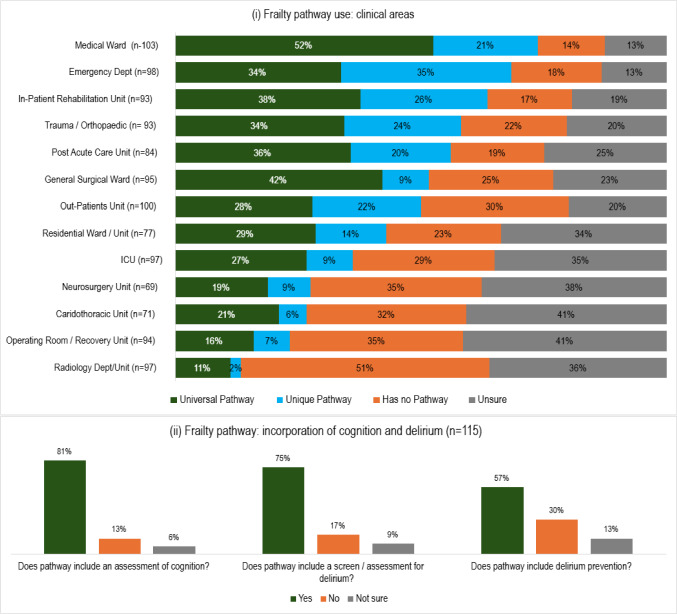
Frailty pathway: **i** prevalence in different clinical areas, **ii** incorporation of cognitive assessment and delirium (screening/assessment and prevention)

A universal frailty pathway was most prevalent in medical (52%) and general surgical (42%) units, followed by inpatient rehabilitation units (38%). Clinical areas most likely to use a unique frailty pathway were the ED (35%) and inpatient rehabilitation units (26%). Having no pathway was most often reported for radiology departments (51%), operating room/recovery units, and neurosurgery units (both 35%). Uncertainty on frailty pathway existence was 13–41%. Respondents whose clinical site had a frailty pathway (currently in existence or in an advanced stage of development) were asked if the pathway incorporated cognitive assessment or delirium screening/prevention. In most cases (81%), an assessment of cognition was incorporated. Screening for delirium was often included (75%). However, delirium prevention was less common (57%) and had the highest rates of omission (30%) and respondent uncertainty (13%). See Fig. 5.

Open-text box comments on the pathways were few and these mainly clarified or reinforced a response, limiting the value of the data. Two respondents gave examples of the variability between dementia pathway used between wards. Some respondents stated a need for training (*n* = 4) and more staff (*n* = 5). Suggestions for improvement included pathway development (*n* = 2) and promoting geriatrics and dementia, delirium, and frailty (*n* = 3).

### Availability of specialist staff or teams to support care

Respondents were asked if they had any specialists operating at their clinical site (ref. Online Resource 2). The availability of specialist staff correlated with the use of dementia, delirium, and frailty pathways. Where specialist staff existed, these were most commonly individual doctors and specialist nurses rather than teams (of two or more specialties). Dementia-specific doctors and nurses were the most reported specialists, especially in Ireland, the UK, and Norway. Respondents reporting highest rates for team specialists were based in the UK, Ireland, Spain, Turkiye, Netherlands, and Belgium, while ten countries reported no specialist teams. Dementia–delirium doctors, nurses, and teams were more frequent than combined dementia–frailty or delirium–frailty roles.

There were 66 responses about dedicated specialist staff. In total, ten respondents clarified that they had been forced to select a response indicating some specialist staff, as there was no option for a “none” response for this specific question. We, thus, removed their data from the count of specialist staff. However, others (*n* = 15) had selected specialist roles, but their open-text responses indicated that these were more likely generic geriatrician and older person nurse roles, e.g., “geriatric liaison service”; “geriatric nurse”; “I can manage any of these conditions… does this mean I am a ‘lead doctor’?”; “geriatricians review delirious/demented patients under other services as needed”; “we help the nurses without much knowledge of dementia or delirium”. Three others referred to outpatient memory clinics, but it was not clear if this was additional to dedicated inpatient services. These responses were not removed from the count, but they indicate that the reported dedicated staff roles (i.e., dedicated staff with specialized training and specific time) may be over-reported in our sample. Five respondents spoke of insufficient staff numbers and staff hiring issues, e.g., “grossly understaffed”; “poorly upskilled team, with multiple vacancies”. One respondent suggested that smaller hospitals do not need to have specialty teams, i.e., that a generic service could cover all three conditions.

## Discussion

This study examines the prevalence of delirium, dementia, and frailty ICPs in clinical settings in Europe, including the incorporation or linkages between pathways where they exist, or consideration of the other conditions within the pathway. The findings reflect ICPs use from a geriatric medicine perspective only.

Respondents reported overall prevalence rates for dementia ICPs, in existence or in development, of 78%. For dementia, national audits of acute hospitals in Northern Ireland, Ireland, and England and Wales in 2012–2014 found that 0%, 6%, and 87%, respectively, of acute hospitals had an ICP for dementia developed or near-developed [29–31]. Repeat audits in 2019 and 2020 (data not available for Northern Ireland) showed little change for Ireland, with a similar number of pathways in existence but more “planned” (previously 6%, now 21%; total now 27%) [32]. There was a small further improvement for England and Wales with 77% of hospitals having a dementia pathway in place, compared to 36% in 2013, and another 15% in development (total now 92%) [33]. Of note, the national audits had required proof of the pathway, which might account for the lower rates than reported in this survey.

Evidence on the value of dementia ICPs is limited, although an evaluation of a dementia pathway (20 cases pre and 23 post) in an acute hospital in Japan reported the ICP supported caregiver understanding and medical practice, and reduced length of stay and hospital costs, although medical staff felt that the pathway was restrictive and time-consuming [36]. This broadly aligns with the findings in a European study on ICPs generally [8]. An analysis of ICP versus treatment-as-usual (*n* = 55) in an inpatient geriatric psychiatry unit in Canada found patients in the ICP group had lower agitation scores, higher likelihood of early discharge, lower rates of psychotropic polypharmacy, and a lower risk of fall during their hospital stay [34]. For frailty, a US retrospective cohort study of frail older trauma patients found a frailty pathway resulted in lower delirium (odds ratio [OR] 0.44, 95% CI 0.22–0.88, *p* = 0.02) and lower 30-day readmission rates (OR 0.25, 95% CI 0.07–0.84, *p* = 0.02), compared to pre-pathway patients [35].

Delirium is a frequent occurrence in people with dementia in clinical settings (49–57%) [36–39] but not all dementia care pathways in our study incorporated or are linked to delirium screening (only 76%) and delirium prevention (only 73%). A 2024 scoping review found dementia care pathways typically include assessments (cognitive, medical history, physical examination, care needs); pharmacological and non-pharmacological interventions; referral pathways, and/or service directories; family support and education; and care coordination. Evaluation indicated high acceptability but low feasibility for normalization into clinical practice [40]. We would suggest that all dementia care pathways include indicators of delirium and need to screen for delirium, using a suitable tool, to prevent delirium in this high-risk population.

Delirium pathways were common in our study, but rare in operating room/recovery units (30%) which is surprising given that emergence agitation and postoperative delirium are common [41]. This may reflect an underreporting due to the respondents’ discipline, and it is possible that an anesthetist or interventionist in the setting might have been aware of a pathway where the geriatrician was not. Although the prevalence of delirium is high in EDs (8–17%) and ICU (7–50%) [37], delirium pathways were not universal in these units. It was surprising that cardiothoracic units did not often implement unique delirium pathways (8%), given postoperative delirium incidence rates of 11.3% to 51.6% in cardiovascular surgery [42]. Similarly in neurosurgery units, the rarity of a unique pathway (7%) contrasts with the high risk (32% in one study) [43], and the assessment of delirium post neurosurgery would be very different to the assessment of delirium on a general medical or surgical ward. In contrast, trauma/orthopedic wards more often had a unique pathway (19%), reflecting the known high rates of delirium post-hip fracture repair [44].

Delirium can unmask existing cognitive impairment and dementia in a third of older people admitted to hospital [45], and a delirium occurrence increases the risk of future dementia and the rate of cognitive decline [46]. Despite the evidence on delirium–dementia linkages, only 62% of delirium care pathways contained guidance on seeking a formal diagnosis of dementia, if not already made. This indicates missed opportunities to detect mild cognitive impairment and early dementia. The NICE clinical guideline on delirium recommends an assessment of cognitive impairment and/or dementia when people first present to hospital, and a follow-up assessment for possible dementia when delirium does not resolve, noting the difficulty in distinguishing between these conditions and the possible existence of both [47]. Any care pathway that includes delirium screening and/or assessment should include, or link to, a dementia diagnostic pathway, or to a delirium follow-up service, so that cognition can be reassessed after an interval, and collateral history of preceding cognitive function can be revisited. An evaluation of an acute hospital frailty pathway in Ireland, which incorporated comprehensive geriatric assessment (CGA) and recommended referral to a memory service for any older patient with a 4AT score of 1 + who did not have an existing diagnosis of a cognitive impairment, reported that 74% of patients with delirium (4AT 4 +) and 61% with possible cognitive impairment (4AT 1–3) without existing diagnoses were referred onward to memory services. Thus, for 2,100 patients screened for frailty, 495 underwent CGA and 248 of these had an abnormal 4AT; the ICP referral requirement led to 91 patients being directed to a memory service who may not otherwise have been identified [48].

Frailty care pathways were less common overall than dementia or delirium care pathways, and were surprisingly not more common in residential, post-acute care, and rehabilitation settings, than on general medical and surgical wards, even though the population in the former would typically be older and frailer. Where frailty ICPs existed, they usually incorporated cognitive assessment (81%) or delirium screening/assessment (75%). However, just over half (57%) included delirium prevention. This is surprising as hospitalized older adults with frailty have a greater risk of delirium [20, 21]. Unfortunately, dementia and delirium ICPs did not always indicate a need or mechanism for frailty screening (61% and 46%, respectively), even though frailty is common in dementia and frail patients have an almost threefold incremental risk of delirium [20]. It must be noted that CGA incorporates much more than just dementia–delirium–frailty, so that issues like nutrition, continence, medication burden, and psychological and social issues must also be assessed and addressed, and all ICPs should align to the principles of CGA.

Despite being developed by a team of geriatricians with expertise in dementia, delirium, and frailty—and having undergone a pilot phase prior to distribution—this study has certain limitations. Data on the number of geriatricians is not readily available for all countries, making it difficult to ascertain a survey response rate. However, the total number of responses was limited, with a median of seven per country, which affects the overall representativeness of the data. This may relate to one or more of several factors, such as increasing clinical workload, survey fatigue, or lack of interest in the topic. Participation was voluntary, which may introduce selection and gender bias, as the relatively small respondent group might not accurately reflect the broader geriatrician population. It is notable that 41% reported a mixed clinical–academic role, as ICPs may be more frequent in university-affiliated hospitals and/or respondents may be more aware of the value of ICPs. Furthermore, although the survey was circulated through professional networks, participants’ adherence to the stated inclusion criteria was self-reported and not independently verified. In some European countries, geriatric medicine is not a defined specialty; thus, responses may have been lower with potential respondents deeming themselves ineligible. Some countries are relatively overrepresented, while Eastern Europe was underrepresented. As country-level responses ranged from 1 to 31 participants (median = 7) across 27 countries, data were reported at an individual rather than a country level.

Although available in 12 European languages via an embedded link, the survey required responses in English, which may have deterred participation. In addition, some geriatricians may not have been fluent in any of the provided languages. For Eastern Europe, a translation was only available in Ukrainian. These factors limit the survey’s validity and inclusivity, as the language bias may have reduced participation and thus data representativeness. Finally, when assessing specialist staff availability (ref. Online Resource 2), in error, no option was given for “none” and while we removed answers from the ten respondents who clearly indicated that they were forced to select a positive option to proceed with the survey, the data may still overrepresent current staffing. In addition, open-text comments from others indicate that there was large variation in what constituted “specialist staff”, so that the data on specialist staff give an idea of trends only. Finally, this survey represents data as reported by geriatricians and it is possible that they were not aware of a pathway in use or in development in certain services. Thus, ICP prevalence in certain hospital areas where geriatricians are less involved, such as the radiology department or an operating theatre’s recovery room, may be underrepresented.

## Conclusion

This pan-European survey provides an overview on the prevalence and interconnectedness of dementia, delirium, and frailty ICPs in clinical settings. Dementia and delirium pathways are common in clinical settings except for radiology, neurosurgery, and operating/recovery rooms. These pathways more often include assessment of the other condition than containing a link to a separate pathway. Frailty pathways are less common overall; similar to dementia and delirium pathways, rates are notably low in radiology and operating/recovery rooms, but also cardiothoracic units. Specific frailty staff are also less common than dementia or delirium staff. However, dementia and to a lesser extent delirium pathways generally incorporate frailty screening which may help offset a more specific focus. Given the known associations between dementia, delirium, and frailty, these findings suggest a need for more joined up thinking to maximize the value of staff effort, reduce duplications, and avoid a siloed approach to the care of older people.

## Supplementary Information

Below is the link to the electronic supplementary material.

Supplementary file1 (DOCX 24 KB)

Supplementary file2 (DOCX 209 KB)

Supplementary file3 (DOCX 38 KB)

## Data Availability

For the purpose of open access, the authors have applied a Creative Commons Attribution (CC BY) license to any author-accepted manuscript version arising from this submission. The complete survey dataset will be available in Open Science Framework from December 2025 onwards.
