# Supplementary material for: Prevalence and interconnectedness of delirium, dementia, and frailty pathways in clinical settings: a survey of geriatricians across Europe
Source: Eur Geriatr Med. 2025 Dec 13;17(2):537–48. doi: 10.1007/s41999-025-01375-w (PMC13109260; doi:10.1007/s41999-025-01375-w)
Supplement: Supplementary file 1 — Supplementary file1 (DOCX 24 KB) [file 41999_2025_1375_MOESM1_ESM.docx]

**Online Resource 1: Supplementary material showing respondent demographics, specialist staff available in clinical sites mapped by country, and survey questionnaire**

Table S1 Respondent demographics (n=240)

| **Region, country** | | **No.** | **Proportion** | **Subtotal** |
| --- | --- | --- | --- | --- |
| **Eastern Europe** | Bulgaria | 1 | 0.4% | 12 |
|  | Czechia | 2 | 0.8% |  |
|  | Poland | 5 | 2.1% |  |
|  | Romania | 3 | 1.3% |  |
|  | Russia | 1 | 0.4% |  |
| **Southern Europe** | Albania | 0 | 0.0% | 76 |
|  | Greece | 6 | 2.5% |  |
|  | Italy | 20 | 8.3% |  |
|  | Malta | 4 | 1.7% |  |
|  | North Macedonia | 0 | 0.0% |  |
|  | Portugal | 9 | 3.8% |  |
|  | Serbia | 0 | 0.0% |  |
|  | Slovenia | 1 | 0.4% |  |
|  | Spain | 16 | 6.7% |  |
|  | Turkiye | 20 | 8.3% |  |
| **Western Europe** | Austria | 5 | 2.1% | 64 |
|  | Belgium | 9 | 3.8% |  |
|  | France | 11 | 4.6% |  |
|  | Germany | 17 | 7.1% |  |
|  | Luxembourg | 3 | 1.3% |  |
|  | Netherlands | 11 | 4.6% |  |
|  | Switzerland | 8 | 3.3% |  |
| **Northern Europe** | Denmark | 9 | 3.8% | 88 |
|  | Finland | 7 | 2.9% |  |
|  | Iceland | 2 | 0.8% |  |
|  | Lithuania | 1 | 0.4% |  |
|  | Norway | 15 | 6.3% |  |
|  | Republic of Ireland | 31 | 12.9% |  |
|  | Sweden | 2 | 0.8% |  |
|  | UK | 21 | 8.8% |  |
