# Supplementary material for: Prevalence and interconnectedness of delirium, dementia, and frailty pathways in clinical settings: a survey of geriatricians across Europe
Source: Eur Geriatr Med. 2025 Dec 13;17(2):537–48. doi: 10.1007/s41999-025-01375-w (PMC13109260; doi:10.1007/s41999-025-01375-w)

**Online Resource 2:** Specialist staff available in clinical sites (respondents could select multiple options) mapped by country, with pathway data also shown for comparison. A team was defined as two or more staff members with dedicated time for this work. In error, “none” was not given as an answer option for this survey question. Responses (n=230) exclude the ten respondents who clearly indicated they had “no staff” in the linked open text response.


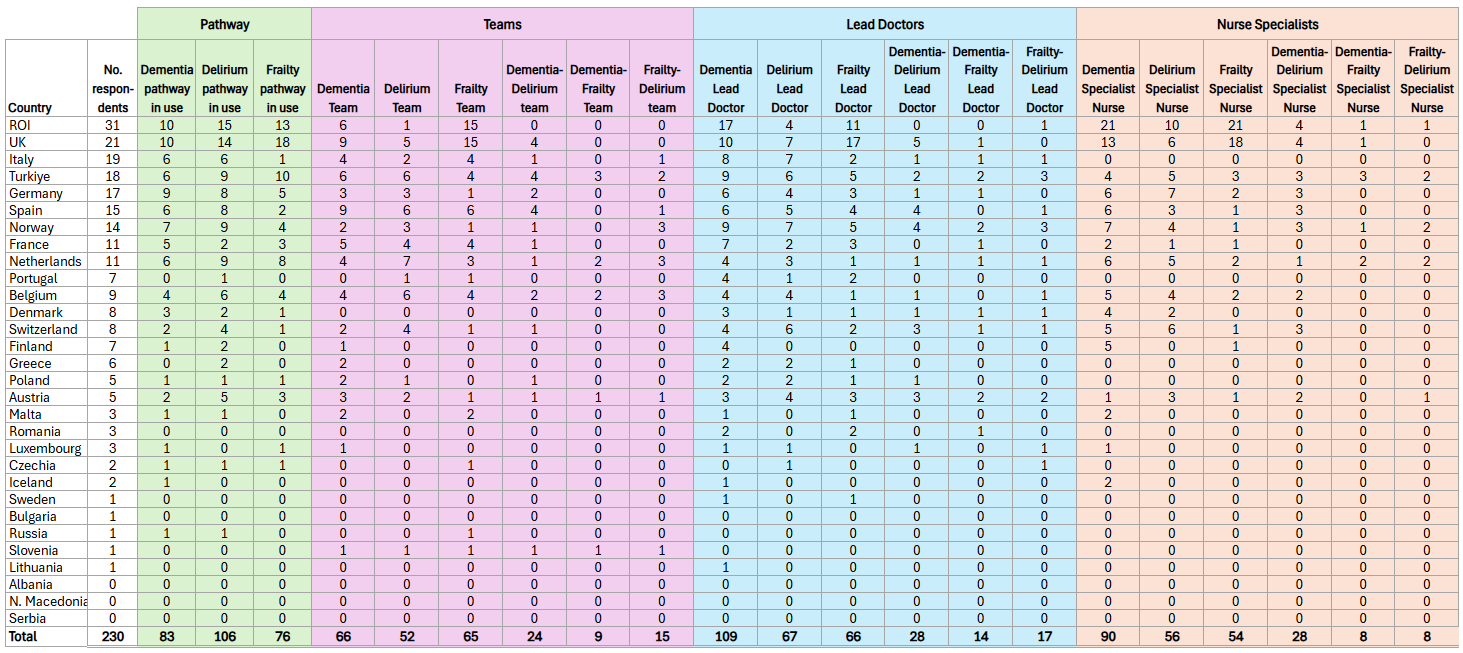

Supplement: Supplementary file 2 — Supplementary file2 (DOCX 209 KB) [file 41999_2025_1375_MOESM2_ESM.docx]
