# Supplementary material for: Prevalence and interconnectedness of delirium, dementia, and frailty pathways in clinical settings: a survey of geriatricians across Europe
Source: Eur Geriatr Med. 2025 Dec 13;17(2):537–48. doi: 10.1007/s41999-025-01375-w (PMC13109260; doi:10.1007/s41999-025-01375-w)
Supplement: Supplementary file 3 — Supplementary file3 (DOCX 38 KB) [file 41999_2025_1375_MOESM3_ESM.docx]

**Online Resource 3: Survey Questionnaire (partial)**

*[Includes: Overview, Consent & Eligibility; Section G. Clinical Care Pathways. Other parts of the overall questionnaire (Sections C-F) are unrelated to this article and therefore not included.]*

**Overview:**Thank you for considering participating in this research project. The following paragraphs explain the project and what your participation would involve, so that you can make an informed choice.

The purpose of this project is to explore the perceptions of geriatricians and senior geriatric trainees in Europe about the complex relationship between dementia, delirium and frailty. This includes relative prevalence, overlaps, causality, etc. The answers you provide will help educators and researchers in all three conditions understand how we perceive these conditions, and particularly where there may be misperceptions or differences in views.

Should you choose to participate, you will be asked to complete a short online, anonymous survey, which will take about 6-12 minutes, depending on your clinical site. Not all questions are mandatory and some important questions come towards the end, so please try to complete the questions at the end before submitting. We have also translated the survey into several European languages if you have difficulty understanding any of the words in the survey. Translations can be accessed by clicking on the following link: **Survey Translations**. Please make sure to answer all survey questions in English only.

Participation in this study is **completely voluntary**. Should you choose to take part, you can skip some questions, or you can decide to end the survey without submitting it. Once you submit your survey, the data cannot be retrieved by us, as it is anonymous.

IP addresses will not be collected at any point, meaning the data you provide cannot be traced back to you. The anonymous data will be stored on the University College Cork OneDrive system and subsequently on a password-protected computer. The pooled data will be stored for a minimum of ten years (as required by our university research data storage policy).

The information you provide may contribute to research publications and/or conference presentations. We do not anticipate any negative outcomes from participating in this study.

This study is unfunded. It has obtained ethical approval from the UCC Social Research Ethics Committee (SREC). If you have any queries about this research, you can contact the postdoctoral researcher, Catriona Curtin. This principal investigator of this survey is Professor Suzanne Timmons.

**Consent:**

If you agree to take part in this survey based on the above information, please indicate your consent below. If you tick ‘yes’, you will be brought to the eligibility check for the survey. Do you consent to participate in this survey?

□ Yes

□ No

**Eligibility:**

Thank you for agreeing to take part in this survey. Please confirm your eligibility by ticking one of the following answers:

□ I am a fully qualified consultant geriatrician (temporary or permanent) working in a European country, currently or within the last 12 months

□ I am a geriatric trainee in my final two years of specialist (higher) geriatric training, based in any European country

□ I am a geriatrician who has retired within the previous three years from working in any European country

□ I do not fit any of the above categories and so I am not eligible for this study

**Section A: Demographics**

Q1. Please select from the following drop down menu, your country of current/recent work.

Albania (1) ... Other (47)

Q2(i). Role: Which of the following relates to you in relation to your current/recent role? Please select one from the following from the drop-down list.

Permanent (1) Temporary (2) Retired (3) Trainee second last year (4) Trainee last year (5)

*Skip To: Q4 If Q2(i). Role: Which of the following relates to you in relation to your current/recent role? Pleas... = Trainee Second Last Year*

*Skip To: Q4 If Q2(i). Role: Which of the following relates to you in relation to your current/recent role? Pleas... = Trainee last year*

Q2(ii). If you are **working or retired**, which of the following is applicable to you in relation to your current or previous (if retired) role. Please select one from the following from the drop-down list:

Fully Clinical (1) Fully Academic (2) Mixed (Both Clinical and Academic Work) (3)

*Display This Question:*

*If Thank you for agreeing to take part in this survey. Please confirm your eligibility by ticking on... != I am a geriatrician who has retired within the previous three years from working in any European country*

Q3. Please select from the following drop-down list, how many years you have worked as a consultant. Please select one response only.

1-5 Years (1) 6-10 Years (2) 11-15 Years (3) 16-20 Years (4) 21-25 Years (5) 25-30 Years (6) 31-35 Years (7) 35+ Years (8)

Q4. Setting: What is or was the nature of your current or most recent workplace setting(s). Please select from the following all the apply.

□ Acute

□ Community

□ Residential

□ Post-Acute

□ Rehabilitation

□ Outpatient Clinic

□ Academic

Q5. Gender:  What is your gender? Please select one of the following.

Male (1) Female (2) Non-binary /Other (3) Prefer not to say (4)

Q6. Please select your special interests from the following list of options, which are arranged as per the EuGMS Special Interest Groups. (You do not need to be a member of that SIG to select the option). Please select all that apply. [list of EuGMS SIG areas given]

Q7. Have or had (if retired) you a lead role in your setting or region/country in any of the following areas? Please select all that apply. [List of EuGMS SIG areas given]

**Section B: Definitions**

Dementia is defined as per DSM-5 criteria.

Delirium is defined as per DSM-5 criteria.

Delirium superimposed on dementia (DSD) is where a person with an underlying dementia develops delirium.

Frailty is understood as a (potentially reversible) clinical syndrome of gradual loss of reserve over time, with increased vulnerability to stressors, leading to functional impairment and adverse health outcomes.

**Section G: Clinical Care Pathways**

*Eligibility*

The last part of the survey deals with dementia, delirium and frailty pathways in a hospital or residential care setting and will take only 3-4 minutes. If have not recently worked in a hospital or rehabilitation or post acute care or residential setting (i.e. you only work in the community or in an academic role), you have completed the survey now.

Please select which of the following is applicable to you to be routed to the appropriate path.

□ I am familiar with a hospital or rehabilitation or post acute care or residential setting.

□ I do not (currently or recently) work in a hospital or rehabilitation or post acute care or residential setting (i.e. I only work in the community or in an academic role).

A clinical care pathway, whether paper-based or electronic, aims to organise and standardise care processes for a specific clinical problem, procedure or episode of care in a specific population, to improve patient outcomes and organisation efficiency.
It is a structured multidisciplinary care plan, used to channel the translation of guidelines or evidence into local structures. It details the steps in a course of treatment/care in a **‘plan’, ‘pathway’, ‘algorithm’, ‘guideline’, ‘protocol’**or other **‘inventory of actions’** (i.e. the intervention has time frames or criteria-based progression). A simple description is that it specifies **which** patients should receive **what** care, by **whom** and **when.** An example might be that all patients over 65 should be screened for delirium on admission to hospital by the admitting nurse, and if positive, [XYZ] should happen (by xyz) within X hours, while if negative [ABC] should happen (by abc) within Y hours.
Sometimes the term “care bundle” is also used- this can be taken to be equivalent to a care pathway.

Q1(i). Has your site a specific care pathway for people with **dementia**?

□ Yes

□ No

□ In development

□ Not sure

Q1(ii). Please indicate below which clinical areas have a dementia pathway in place or development (it does not matter if it is the same pathway in all areas, or different versions for different wards/units):

|  | **Not available in my clinical site** | **Uses an Overall Pathway** | **Uses a unique Pathway** | **Has no Pathway** | **Unsure** |
| --- | --- | --- | --- | --- | --- |
| Medical Wards |  |  |  |  |  |
| Surgical Wards |  |  |  |  |  |
| Trauma/Orthopaedic Ward |  |  |  |  |  |
| Emergency Department |  |  |  |  |  |
| Intensive Care Unit |  |  |  |  |  |
| Radiology Department |  |  |  |  |  |
| Operating Room/Recovery Unit |  |  |  |  |  |
| Neurosurgery Unit |  |  |  |  |  |
| Cardiothoracic Unit |  |  |  |  |  |
| Out-Patients Unit |  |  |  |  |  |
| In-Patient Rehabilitation Unit |  |  |  |  |  |
| Post Acute Care Unit |  |  |  |  |  |
| Residential Ward/Unit |  |  |  |  |  |

Q1(iii). **If you have a dementia pathway or a dementia pathway is in an advanced state of development,** where you mainly work in this site: Does this incorporate delirium screening?

□ Yes, embedded within it

□ Yes, clear links to a separate delirium screening pathway

□ No

□ Not sure

Q1(iv). Does this incorporate delirium prevention?

□ Yes, embedded within it

□ Yes, clear links to a separate delirium prevention pathway

□ No

□ Not sure

Q1(v). Does this incorporate frailty screening/assessment?

□ Yes, embedded within it

□ Yes, clear links to a separate frailty pathway

□ No

□ Not sure

Please qualify or clarify any answer if you wish:

________________________________________________________________

________________________________________________________________

Q2(i).  Has your site a specific care pathway for people with **delirium**?

□ Yes

□ No

□ In development

□ Not sure

Q2(ii). Please indicate below which clinical areas have a **delirium** pathway in place or development (it does not matter if it is the same pathway in all areas, or different versions for different wards/units):

|  | **Not available in my clinical site** | **Uses an Overall Pathway** | **Uses a unique Pathway** | **Has no Pathway** | **Unsure** |
| --- | --- | --- | --- | --- | --- |
| Medical Wards |  |  |  |  |  |
| Surgical Wards |  |  |  |  |  |
| Trauma/Orthopaedic Ward |  |  |  |  |  |
| Emergency Department |  |  |  |  |  |
| Intensive Care Unit |  |  |  |  |  |
| Radiology Department |  |  |  |  |  |
| Operating Room/Recovery Unit |  |  |  |  |  |
| Neurosurgery Unit |  |  |  |  |  |
| Cardiothoracic Unit |  |  |  |  |  |
| Out-Patients Unit |  |  |  |  |  |
| In-Patient Rehabilitation Unit |  |  |  |  |  |
| Post Acute Care Unit |  |  |  |  |  |
| Residential Ward/Unit |  |  |  |  |  |

Q2(iii). **If you have a delirium pathway or a delirium pathway is in an advanced state of development,** where you mainly work in this site: Does this incorporate directions or guidance about obtaining a formal dementia diagnosis where a patient is felt to have an underlying undiagnosed dementia?

□ Yes, directions/guidance are embedded within the pathway

□ Yes, clear links to a separate dementia diagnostic pathway

□ No

□ Not sure

Q2(iv). Does this delirium pathway differentiate between the management of delirium and delirium superimposed on dementia (DSD)?

□ Yes

□ No

□ Not sure

Q2(v). Does this delirium pathway incorporate frailty screening/assessment?

□ Yes, embedded within it

□ Yes, clear links to a separate frailty pathway

□ No

□ Not sure

Please qualify or clarify any answer if you wish:

________________________________________________________________

________________________________________________________________

|  |
| --- |

Q3(i). Has your site a specific care pathway for people with **frailty**?

□ Yes

□ No

□ In development

□ Not sure

Q3(ii). Please indicate below which areas have a frailty pathway in place or development (it does not matter if it is the same pathway in all or different versions for different wards/units):

|  | **Not available in my clinical site** | **Uses an Overall Pathway** | **Uses a unique Pathway** | **Has no Pathway** | **Unsure** |
| --- | --- | --- | --- | --- | --- |
| Medical Wards |  |  |  |  |  |
| Surgical Wards |  |  |  |  |  |
| Trauma/Orthopaedic Ward |  |  |  |  |  |
| Emergency Department |  |  |  |  |  |
| Intensive Care Unit |  |  |  |  |  |
| Radiology Department |  |  |  |  |  |
| Operating Room/Recovery Unit |  |  |  |  |  |
| Neurosurgery Unit |  |  |  |  |  |
| Cardiothoracic Unit |  |  |  |  |  |
| Out-Patients Unit |  |  |  |  |  |
| In-Patient Rehabilitation Unit |  |  |  |  |  |
| Post Acute Care Unit |  |  |  |  |  |
| Residential Ward/Unit |  |  |  |  |  |

Q3(iii). **If you have a frailty pathway, or a frailty pathway is in an advanced state of development,**where you mainly work in this site:

|  | **Yes** | **No** | **Not sure** |
| --- | --- | --- | --- |
| i) Does this pathway include an assessment of cognition? |  |  |  |
| ii) Does this pathway include a screen/assessment for delirium? |  |  |  |
| iii) Does this pathway include delirium prevention? |  |  |  |

Please qualify or clarify any answer if you wish:

________________________________________________________________

________________________________________________________________

Q4(i). Does your clinical site have any of the following: (Tick as many as apply). *(A team is 2 or more staff disciplines with dedicated time for this work)*

□ Dementia Specialist Nurse

□ Dementia Lead Doctor

□ Dementia Team

□ Delirium Specialist Nurse

□ Delirium Lead Doctor

□ Delirium Team

□ Frailty Specialist Nurse

□ Frailty Lead Doctor

□ Frailty Team

□ Dementia-Delirium Specialist Nurse

□ Dementia-Delirium Lead Doctor

□ Dementia-Delirium Team

□ Frailty-Delirium Specialist Nurse

□ Frailty-Delirium Lead Doctor

□ Frailty-Delirium Team

□ Dementia-Frailty Specialist Nurse

□ Dementia-Frailty Lead doctor

□ Dementia-Frailty Team

Q4(ii). Please give details of the composition of the service or team, and any comments you wish here:

________________________________________________________________

Q4(iii). Have you any other comments on how dementia-delirium-frailty is handled in your site or how care could be improved?

________________________________________________________________

End
